# Book Reviews

**Published:** 1892-03

**Authors:** 


### Book Reviews.

---

Physicians' Leisure Library. Pulmonary Consumption a Nervous Disease, Considered as such from a Practical, a Clinical, and a Therapeutical Standpoint. By Thomas J. Mays, M. D., Professor of Diseases of the Chest in the Philadelphia Polyclinic and College for Graduates in Medicine; Visiting Physician to the Rush Hospital for Consumption, of Philadelphia; Member of the Philadelphia County Medical Society; Member of the Neurological Society of Philadelphia, etc., etc. Detroit, Mich.: George S. Davis, Detroit, Mich. 1891.

This essay is an attempt to substitute for the bacillus theory one showing that the origin of consumption is in the nervous system.

The argument is based on these facts: first, that the children of consumptives are often hysterical; second, that many cases of pulmonary disease have been associated with diseases of the vagi, with bulbar paralysis, disease of the medulla, tabes dorsalis, and multiple neuritis; third, that there is a relationship between alcoholism and consumption; fourth, that there is a relationship between syphilis and pulmonary consumption. These last two causes—syphilis and alcohol—he maintains, operate because they both have a special affinity for the nervous system, and produce the lung lesions through the pneumogastric nerves. Fifth, twenty-four post mortems on epileptics have shown pulmonary disease, and disease either of the vagi or of the medulla oblongata. Sixth, that diabetes, also beri-beri, leprosy, lupus, and pellagra have occurred in the same patients.

The statement that the children of consumptives are often hysterical is true, but we do not see that that warrants the deduction that the nervous disorder in one generation is a proof that the consumption in the parents was a nervous disease. It simply proves to us that invalid persons cannot expect healthy, vigorous children. The children of the aged are not vigorous.

That alcoholism is frequently found in the ancestry of consumptives is a sad fact. But it is sadder still to think that alcoholism is so general and so widespread that we cannot think of any disease that afflicts mankind among whose victims we will not find a certain proportion with an alcoholic ancestry.

We can say of syphilis and consumption that we do not deny the fact that they both affect the pulmonary tissues. We will go farther, and say that they both affect bone, cutaneous and nerve tissue. To us it proves that the germ of these two diseases can flourish in many different soils.

We come now to the question of the relation of epilepsy and consumption. The author starts out with the statement that Vander Kolk, Marshall Hall, Brown-Sequard, Kussmaul and Zenner point out conclusively that the chief seat of epilepsy is located in the medulla oblongata. This statement we deny. They did not prove this *conclusively*, as is shown by the fact that Rosenbach proved that stimulation of the medulla caused tonic and not clonic spasms. He denies the possibility of the medulla being the epileptic center. Opposed to the theory which the author says is conclusively proven, we find such noted experimenters and authorities as Sepilli, Albertoni, Ferrier, Luciani, Bartholow, Unverreicht, Munk, Budnow, Heidenhain, Horsley, and Hare. Thus we see that the corner-stone on which the author is building his fabric is not resting on the solid foundation he would have us believe.

That phthisis and epilepsy may occur in the same patient, no one will deny. So may phthisis and barbers' itch. We do not believe that the coexistence of two diseases in one patient proves that they have the same origin.

The book closes with a description of the treatment of phthisis according to the theory of the nervous origin of the disease. We had expected in this chapter to find something new, but, on the contrary, we find our old friends. *Rest* for those who become tired on exertion. If they are able, let them walk out and sit in the open air. If not able to walk out, let them be carried out. Antipyrin, antifebrin, and phenacetin for high temperature. A dietary of the most nutritious foods, cod-liver oil, hypophosphites, pulmonary gymnastics, inhalation of oxygen, nitrous oxide, and compressed air.

We find galvanization of the pneumogastric nerves, and painting with iodine along their course recommended. But we find nothing about the results of this treatment. We are left to infer what we please.

The theory of the author is an interesting one, and as we read it we wished we might be convinced, but the weight of evidence is against it, and in our opinion the author has not proved his case.

J. W. P.

---

MASSAGE, AND THE ORIGINAL SWEDISH MOVEMENTS. Their Application to Various Diseases of the Body. Lectures before the Training Schools for Nurses connected with the Hospital of the University of Pennsylvania, German Hospital, Woman's Hospital, Philadelphia Lying-in Charity Hospital, and the Kensington Hospital for Women, of Philadelphia. By KURRE W. OSTROM, from the Royal University of Upsala, Sweden; Instructor in Massage and Swedish Movements in the Philadelphia Polyclinic and College for Graduates in Medicine. Second edition. Enlarged, with eighty-seven illustrations. Philadelphia: P. Blakiston, Son & Co., 1012 Walnut street. 1891.

So much attention has been given to the so-called Swedish movement and massage of late, that its literature is fast becoming redundant. In reality we see but little difference as to the methods of their professors. Based on the anatomical structure of the body, the intelligent use of either, or both, must be substantially alike.

Ordinary gymnastic exercises aim to give the human frame its most harmonious development, while massage is supposed to be mostly confined to the sick room, and consists mainly in an intelligent manipulation in the line of the course of the blood-vessels, or kneadings and other uses of the hand in starting sluggish capillaries.

While readily admitting the therapeutic value of massage when intelligently applied, we cannot but fear that the author, in common with other advocates of the system, is too sanguine in regard to its advantages in cases of diseases of the internal organs, especially those which by their anatomical positions and relations would seem to be beyond external manipulation, except, perhaps, in cases of emaciated patients, where absence of fat and muscular tonicity may enable him to effect the part.

In regard to the value of the so-called Swedish movement, whether curative or prophylactic, and which forms the second and, perhaps, more important part of the treatise, it is not too much to say that it addresses itself directly to the reason of the physiologist, and that it is intelligently exhibited by Mr. Ostrom in his little work. One advantage in this form of health-giving gymnastics lies in its applicability to sick and well alike, and that it does not require absence of clothing. By proper and scientific movements of the joints and muscles the flow of blood is accelerated, the reflex action of the nervous system increased, the stimulating effect of which will be felt throughout the whole frame.

So far as we may judge, Mr. Ostrom's directions are clear and those of a man thoroughly acquainted with his subject and its practical application. The illustrations are simple and easily understood, the typography excellent, and the whole forms a handy little book of 143 pages.

---

Age of the Domestic Animals. Being a Complete Treatise on the Dentition of the Horse, Ox, Sheep, Hog, and Dog, and on the Various Means of Determining the Age of these Animals. By Rush Shippen Huidekoper, M. D., Veterinarian (Alford, France); Professor of Sanitary Medicine and Veterinary Jurisprudence, American Veterinary College, New York; Lieutenant-Colonel and Surgeon-in-Chief National Guard of Pennsylvania; Fellow of the College of Physicians, Philadelphia; Honorary Fellow Royal College of Veterinary Surgeons, London; late Dean of the Veterinary Department, University of Pennsylvania, etc. Illustrated with 200 engravings. Octavo, pp. 212. Philadelphia and London: F. A. Davis, publisher. 1891.

This book, judging from the moderately critical attention we have been able to bestow upon it, seems to have the merit of fulfiling the promise of its title. The matter of which it treats is one of great importance in a certain sense to all, since we all are so largely dependent on our domestic animals, but especially so to the farmer, to the stock raiser, and, indeed, to all whose business or pleasure requires the possession of domestic animals.

Although the scope of the book takes in the dentition of other domestic animals, the part of most general interest to the ordinary reader will be found in what relates to the horse, since not only is he, in some sense, man's most useful companion, but it is also the animal whose age is most commonly and most surely determined by the state of his teeth ; hence, it is this part of the subject which forms by far the largest part of the treatise.

Dr. Huidekoper says in his introduction :

We judge of an animal from its general aspect, from the various changes in the conformation of the body, both external and internal, and from the functional activity of its various organs. All of these are to be examined in detail, and the synthesis of the result usually gives an accurate indication of its age. The most important details in judging of the age, especially in the horse, is found in the teeth." * *

And again he says: "In the ox and sheep, again, the teeth are of good value in judging of age, but the epidermic products, in the shape of the horns, are also important factors after they have become adults." In the dog he considers the teeth are valuable in indicating the age only in young animals ; but in older ones, owing to these modifying circumstances, alterations of the epidermic appendages and general aspect must be our guide.

A full account of the dentition, the growth, form, and histology of the teeth of the horse forms a considerable part of the work. Attention is paid to the duration of the life of the animal, and principles for determining its age and character by the teeth are noted. The thirty years which constitute the life of a horse are divided into five successive periods, showing the state of the teeth in each period, and the normal changes by which one may judge. Irregularities are then described and illustrated, as is indeed every part of the work by finely executed wood-cuts, after which the author turns his attention to the cow, the sheep, the goat, the pig, and, last of all, to the dog.

The book is well written and, as above remarked, well illustrated, and makes interesting reading, to say nothing of its general usefulness.

---

A B C OF THE SWEDISH SYSTEM OF EDUCATIONAL GYMNASTICS. A Practical Hand-Book for School Teachers and the Home. By HARTVIG NISSEN, Instructor of Physical Training in the Public Schools of Boston, Mass.; Instructor of Swedish and German Gymnastics at Harvard University's Summer School, 1891 ; formerly Instructor of Physical Culture at the Catholic University, Washington, D. C., etc., etc. With seventy-seven illustrations. Philadelphia and London : F. A. Davis, publisher. 1891.

Gymnastic exercises aim to give the human frame its most harmonious development in every part, so that, like the good deacon's one-horse *shay*, it shall equally bear the wear and tear — whether of mind or body. Any exercise that develops abnormal strength of a part or parts of the body transcends health requirement. It is pleasant to find that tendency of the system taught and advocated in Mr. Nissen's little volume, is wholly within the scope of a proper and intelligent physical culture, and cannot other than be a valuable auxiliary to the school in the training of the young. We speak of a healthy mind in a healthy body as if they were separate parts of man. A well-balanced brain can hardly exist in a feeble frame, since its strength and well-being must depend on the circulatory system, and it is just that which a proper line of gymnastics sets agoing with the greatest likelihood of success.

The work is systematically arranged in the form of a catechism, with the view to the convenience of the teacher, and profusely illustrated by cuts, which in their simplicity and clearness leave nothing to be desired.

---

THE MICROSCOPE AND ITS REVELATIONS. By the late WILLIAM B. CARPENTER, C. B., M. D., LL. D., F. R. S. Seventh edition; enlarged and revised by the REV. W. H. DALLINGER, LL. D., F. R. S., etc., with twenty-one plates and 800 wood engravings. Octavo, pp. 1099. Philadelphia: P. Blakiston, Son & Co., 1012 Walnut street. 1891.

A standard work like Carpenter's text-book on microscopy needs no introduction to the professional readers, no encomiums and no adulations. By the time it has reached its seventh edition all errors will have been corrected, and the work passed on its merits.

In this new edition, revised by Mr. Dallinger, the first seven chapters have been entirely re-written — in part by those who have been instrumental in bringing these changes about; thus the chapters on the Principles and Theory of Vision with the Compound Microscope were carefully reviewed by Prof. Abbe, of Jena; and so in other departments,— the work has been sublet, so that it is more or less cyclopedic in character.

The opening chapters deal with the theory and construction of the microscope, describing accurately the simple microscope of Galileo, Descartes, Campani, through its various stages, until we reach the beautiful instrument of Zeiss, Beck, Bausch & Lomb, Zentmayer, etc., of the present day. The chapters on Methods of Preparation of Material, Staining, Mounting, etc., show that the editor has been a laborious and conscientious student. The chapters following treat summarily of the whole Animal, Vegetable, and Inorganic Kingdoms. But one chapter of fifty pages is devoted to Animal Histology. For the microscopist working in no special field, perhaps no better work can be recommended, but for the histologist, other books are necessary.

The plates are handsomely executed, and add much to the worth of the treatise. W. C. K.

---

ESSENTIALS OF NERVOUS DISEASES AND INSANITY. Their Symptoms and Treatment. A Manual for Students and Practitioners, by JOHN C. SHAW, M. D. Fifty-eight original illustrations, mostly selected from the Author's Private Practice. Philadelphia: W. B. Saunders, 713 Walnut street. 1891.

This is No. 21 of Saunders' Question Compends, and for the class of book we consider it unusually good. Its bibliographical references are valuable, and the illustrations are well selected. It is too incomplete in its discussion of any disease to be taken as anything more than as a guide to the text-books to which reference is always given. J. W. P.

---

ESSENTIALS OF MEDICAL ELECTRICITY. By D. D. STEWART, M. D., Demonstrator of Diseases of the Nervous System, and Chief of the Neurological Clinic in the Jefferson Medical College, etc., and E. S. LAWRANCE, M. D., Chief of the Electrical Clinic and Assistant Demonstrator of Diseases of the Nervous System, in the Jefferson Medical College, etc. With sixty-five illustrations. Philadelphia: W. B. Saunders, 913 Walnut street. 1892.

This is No. 23 of the Question Compend series, and is written expressly for students. The book contains the usual illustrations of batteries, motor-points, electrodes, etc. There is one decided criticism which we wish to offer, and that is the lack of definiteness of the authors in speaking of the strength of currents and of the duration of time for a sitting. For instance, on page 148, we read that spasmodic wryneck should be treated by a current of moderate strength. A student does not know whether that means five, ten, or twenty milliamperes. No directions are given as to the number of minutes each sitting should last, nor as to the frequency of sittings. These are all important points, and should be mentioned just as certainly as we would direct the dose of a prescribed medicine and the frequency of taking it. The generalities of the book are good. J. W. P.

